# Changes in Mortality According to Creatinine/Cystatin C Ratio in Chronic Kidney Disease and Non-chronic Kidney Disease Patients

**DOI:** 10.3389/fmed.2022.810901

**Published:** 2022-03-02

**Authors:** Jeong Ah Hwang, Younghoon Song, Jaeun Shin, Eunjung Cho, Shin Young Ahn, Gang Jee Ko, Young Joo Kwon, Ji Eun Kim

**Affiliations:** ^1^Department of Internal Medicine, Korea University Guro Hospital, Seoul, South Korea; ^2^Department of Internal Medicine, Korea University College of Medicine, Seoul, South Korea

**Keywords:** creatinine, cystatin C, creatinine/cystatin C ratio, renal dysfunction, mortality

## Abstract

**Background:**

Serum creatinine and cystatin C are not only good indicators of renal function but have also been confirmed to be related to disease prognosis and mortality in various diseases via creatinine/cystatin C ratio (CCR). However, although they are biomarkers of renal function, there is no study regarding renal impairment as a confounding variable in the relationship between CCR and all-cause mortality.

**Methods:**

Patients who had simultaneous measurements of serum creatinine and cystatin C between 2003 and 2020 were enrolled. The patients with chronic kidney disease (CKD) were defined as having an estimated glomerular filtration rate (eGFR) CKD-EPI Cr-Cystatin C < 60 ml/min/1.73 m^2^. CCR was calculated by dividing the serum creatinine level by the cystatin C level measured on the same day. The main outcome assessed was all-cause mortality according to CCR in CKD or non-CKD groups.

**Results:**

Among the 8,680 patients in whom creatinine and cystatin C levels were measured simultaneously, 4,301 were included in the CKD group, and 4,379 were included in the non-CKD group, respectively. CCR was 1.4 ± 0.6 in total participants. The non-CKD group showed higher mean CCR, (1.5 ± 0.7 vs. 1.3 ± 0.5) as well as a wider distribution of CCR (*p* < 0.001) when compared to the CKD group. In non-CKD group, 1^st^, 4^th^ and 5^th^ quintiles of CCR significantly increased the all-cause mortality risk compared to 2^nd^ quintile of CCR, suggesting U-shaped mortality risk according to CCR in non-CKD. On the other hand, in CKD group, the risk of all-cause mortality linearly increased and 5^th^ quintile of CCR showed 1.82 times risk of mortality compared to 2^nd^ quintile of CCR. In the subgroup analysis of mortality by age and sex, the mortality difference according to CCR were diminished in old age and female sex subgroups.

**Conclusion:**

We discovered a U-shaped relationship between mortality and CCR levels in normal renal function, and an increased risk of mortality in CKD with elevated CCR.

## Introduction

eGFR is a rapid and convenient routine method for measuring renal function. Currently, the Chronic Kidney Disease Epidemiology Collaboration (CKD-EPI) equation is the most widely used method in clinical settings among the various equations based on creatinine. However, creatinine has some limitations as an ideal biomarker of renal function. First, serum creatinine concentration does not rise until 50% of the active nephron is damaged, suggesting poor sensitivity to small changes in renal function (1). In addition, because creatinine is constantly generated by muscles in the human body, serum creatinine levels can be affected by large biological variability, including age, sex, nutritional status, and muscle mass (2, 3). These intrinsic factors related to creatinine metabolism may result in inaccurate estimation of renal impairment.

In the context of these limitations, cystatin C has been proposed as another renal biomarker that may replace or compensate for creatinine. Serum cystatin C concentration was less dependent on the factors that affect creatinine. However, cystatin C measurement is expensive, and a recent study reported that it is affected by various chronic inflammatory conditions associated with diabetes mellitus (DM), obesity, and smoking (4–6).

To compensate for the limitations of each biomarker, both can be measured simultaneously. One study suggested higher accuracy of renal function assessment when both biomarkers are measured simultaneously, compared to results obtained when each is measured alone (7). However, because of the difference in their molecular properties, there are some discrepancies between the serum levels of the two biomarkers, which is thought to be related to muscle mass or nutritional status. In this context, several researchers have evaluated the difference between creatinine and cystatin C and tried to find the clinical implication of the discrepancy between the two biomarkers. Previous studies have shown that elevated serum creatinine/cystatin C ratio (CCR) is associated with various diseases, including chronic obstructive pulmonary disease (COPD) (8), DM (9), non-alcoholic fatty liver disease (10), obstructive coronary artery disease (11) and cancer (12, 13) as well as all-cause mortality. Surprisingly, although both cystatin C and creatinine are cleared by renal excretion and are widely used as biomarkers of renal function, there is no study concerning renal impairment as a confounding variable in the relationship between CCR and hard outcomes. Therefore, in this study, all-cause mortality according to CCR was assessed by dividing the patients into two groups: those diagnosed with traditional CKD criteria (i.e., the CKD group) and those who did not meet the traditional CKD criteria (non-CKD group).

## Methods

### Study Subjects

The study was approved by the Institutional Review Board of Korea University Guro Hospital (approval no. 2021GR0523) and complied with the Declaration of Helsinki. Informed consent was waived under the approval of the review board. Among the adult patients who visited outpatient clinics in Korea University Guro Hospital between 2003 and 2020, the patients who measured serum cystatin C and serum creatinine on the same day were included in this retrospective cohort study.

### Data Collection and Definition

We collected demographic characteristics of the participants, including age, sex, and body mass index, as well as underlying diseases, including hypertension, DM, myocardial infarction, and cancer. Laboratory tests, such as hemoglobin, albumin, C-reactive protein (CRP), uric acid, creatinine, cystatin C, and dipstick proteinuria were also performed. All collected data were obtained by reviewing electronic medical records. The CCR was calculated by dividing the serum creatinine level by the cystatin C level measured on the same day. We classified the participants into non-CKD and CKD groups based on their renal function. CKD was defined as eGFR <60 ml/min/1.73 m^2^ calculated by recently revised CKD-EPI Cr-Cystatin C equation (14).

The primary outcome of this study was all-cause mortality according to CCR in non-CKD and CKD participants. Mortality risks were represented as comparisons between quintiles of CCR.

### Statistical Analysis

Continuous variables were expressed as means with standard deviations or medians with interquartile ranges, and categorical variables were expressed as numbers and proportions. For comparison of continuous variables between the two groups, the *t-*test or Mann-Whitney U test was used as appropriate. The chi-square test was used to compare categorical variables between the two groups. To compare the distribution of CCR between the two groups, the Kolmogorov-Smirnov equality-of-distributions test was performed. Univariable and multivariable Cox regression analyses were used to assess mortality risk. A cubic spline curve was generated with the multivariable Cox regression results according to the multiple levels of variables using the mkspline function in Stata. Statistical significance was set at *P* < 0.05. All statistical analyses were performed using Stata version 15.1 (StataCorp. 2017. *Stata Statistical Software: Release 15*. College Station, TX: StataCorp LLC.).

## Results

### Baseline Characteristics

Among the 8,680 participants enrolled, the CKD group consisted of 4,301 participants while the non-CKD group included 4,379 participants. The CKD group showed higher age, a larger proportion of females, and higher percentages of underlying diseases including hypertension, DM, myocardial infarction, and cancer when compared to the non-CKD group. Furthermore, CKD participants had lower albumin and hemoglobin levels, and higher CRP and uric acid levels. Mean eGFR of the CKD and non-CKD groups were 34.3 ± 15.1 and 95.1 ± 22.4 mL/min/1.73 m^2^, respectively (Table 1).

**Table 1:** Baseline characteristics of the study participants.

**Variables**	**Total**	**Non-CKD**	**CKD**	***p-*** **value**
	***n =*** **8,680**	***n =*** **4,379**	***n =*** **4,301**	
Age, years	66.7 ± 15.2	63.4 ± 15.6	70.1 ± 13.9	<0.001
Male sex, *n* (%)	4,956 (57.1%)	1,888 (43.1%)	1,836 (42.7%)	<0.001
BMI, kg/m^2^	25.8 ± 44.3	26.0 ± 52.6	25.6 ± 33.9	0.355
HTN, *n* (%)	5,619 (64.7%)	2,286 (52.2%)	3,333 (77.5%)	<0.001
DM, *n* (%)	4,385 (50.5%)	1,691 (38.6%)	2,694 (62.6%)	<0.001
Myocardial infarction, *n* (%)	277 (3.2%)	98 (2.2%)	179 (4.2%)	<0.001
Cancer, *n* (%)	1,639 (18.9%)	702 (16.0%)	937 (21.8%)	<0.001
Serum albumin, g/dL	3.6 ± 0.6	3.7 ± 0.6	3.5 ± 0.6	<0.001
Hemoglobin, g/dL	11.6 ± 2.3	12.4 ± 2.2	10.8 ± 2.1	<0.001
Dipstick urine protein ≥ 1+, *n* (%)	3,319 (38.2%)	1,188 (27.1%)	2,131 (49.5%)	<0.001
C-reactive protein, mg/L	28.4 ± 60.9	24.1 ± 55.9	32.6 ± 65.2	<0.001
Uric acid, mg/dL	5.7 ± 2.3	4.9 ± 1.9	6.4 ± 2.4	<0.001
eGFR CKD-EPI Cr, ml/min/1.73 m^2^	50.6 ± 30.1	73.4 ± 24.3	27.4 ± 12.6	<0.001
eGFR CKD-EPI Cystatin C, ml/min/1.73 m^2^	71.6 ± 39.6	103.7 ± 25.1	38.9 ± 20.2	<0.001
eGFR CKD-EPI Cr-Cystatin C, ml/min/1.73 m^2^	65.0 ± 35.9	95.1 ± 22.4	34.3 ± 15.2	<0.001
CCR	1.4 ± 0.6	1.5 ± 0.7	1.3 ± 0.5	<0.001

*CKD, chronic kidney disease; BMI, body mass index; HTN, hypertension; DM, diabetes mellitus; eGFR, estimated glomerular filtration rate; CKD-EPI, chronic kidney disease epidemiology collaboration; CCR, serum creatinine/cystatin C ratio*.

**Figure 1:**
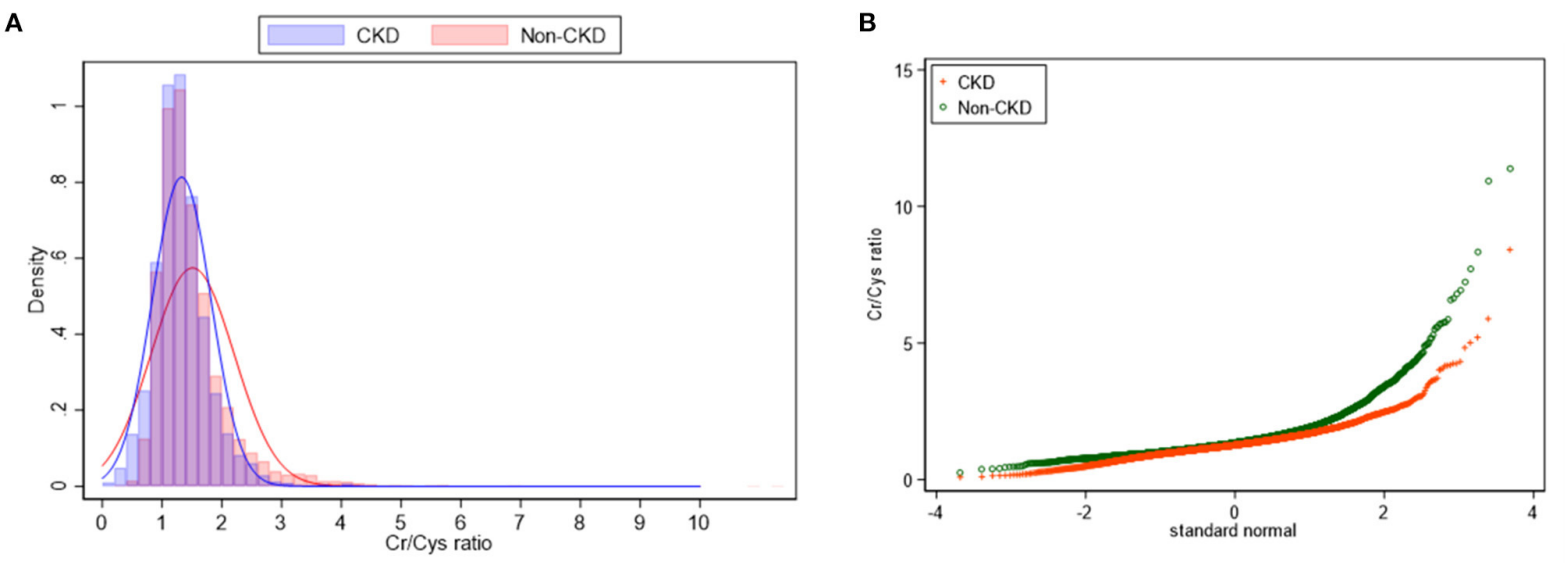
Density plot **(A)** and Q-plot **(B)** for the distribution of CCR between CKD and non-CKD participants.

The CCR of all enrolled participants was 1.4 ± 0.6. The non-CKD group showed a higher mean CCR (1.5 ± 0.7 vs. 1.3 ± 0.5) but a wider distribution of CCR (*p* < 0.001) compared to the non-CKD group (Figure 1).

### All-Cause Mortality According to CCR

**Figure 2:**
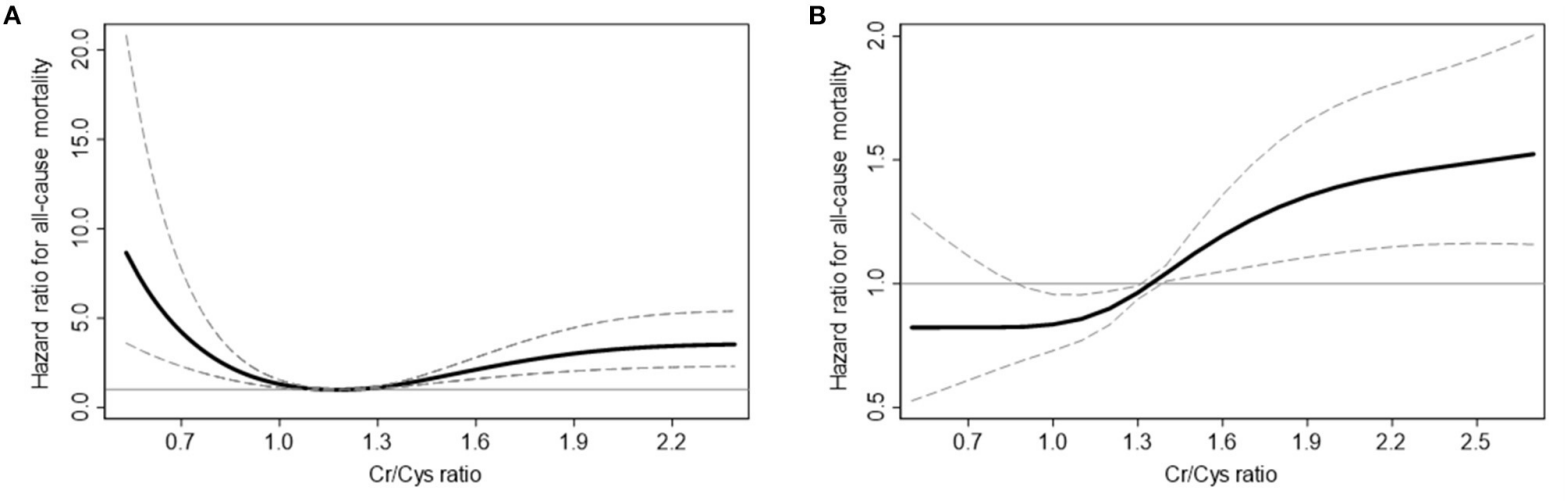
Cubic spline curves for all-cause mortality in non-CKD **(A)** and CKD **(B)** participants. Solid curves represent the hazard ratio for all-cause mortality, and dashed lines represent the upper and lower confidence interval of the hazard ratio.

In the non-CKD group, 363 (8.3%) patients died during 402 (183–706) days. When comparing mortality according to CCR, the non-CKD patients with 2^nd^ quintile of CCR showed lowest mortality risk. Compared to 2^nd^ quintile, 1^st^ quintile and 5^th^ quintile of CCR showed 3.07 and 3.84 times elevated mortality risk in multivariable analyses, respectively (Table 2). While the mortality risk in the non-CKD group showed a U-shape in the spline curve for mortality, the mortality risk in the CKD group was different (Figure 2). In the CKD group, 539 (12.5%) patients died during 440 (192–717) days. The risk of mortality was linearly increased and 4^th^ and 5^th^ quintile of CCR showed 1.46 and 1.82 times increase risk of mortality compared to 2^nd^ quintile in multivariable analyses.

**Table 2:** All-cause morality according to CCR in non-CKD and CKD participants.

**CCR**	**Unadjusted**		Model 1^*^		Model 2^**^	
	**HR (95% CI)**	***p-*** **value**	**HR (95% CI)**	***p-*** **value**	**HR (95% CI)**	***p-*** **value**
**Non-CKD**						
1^st^ quintile	1.76 (0.91–3.43)	0.095	2.25 (1.09–4.62)	0.028	3.07 (1.40–6.75)	0.005
2^nd^ quintile	1 (ref)		1 (ref)		1 (ref)	
3^rd^ quintile	2.31 (1.23–4.33)	0.009	2.35 (1.20–4.60)	0.012	1.87 (0.90–3.88)	0.094
4^th^ quintile	5.00 (2.81–8.88)	<0.001	5.30 (2.85–9.85)	<0.001	2.99 (1.52–5.88)	0.002
5^th^ quintile	14.30 (8.32–24.57)	<0.001	12.86 (7.08–23.35)	<0.001	3.84 (1.97–7.49)	<0.001
**CKD**						
1^st^ quintile	1.44 (1.05–1.96)	0.022	1.56 (1.12–2.17)	0.009	1.12 (0.77–1.64)	0.557
2^nd^ quintile	1 (ref)		1 (ref)		1 (ref)	
3^rd^ quintile	1.28 (0.92–1.78)	0.144	1.26 (0.90–1.78)	0.179	1.37 (0.94–2.00)	0.099
4^th^ quintile	1.90 (1.39–2.58)	<0.001	1.85 (1.34–2.56)	<0.001	1.46 (1.02–2.09)	0.040
5^th^ quintile	3.61 (2.69–4.85)	<0.001	3.41 (2.48–4.69)	<0.001	1.82 (1.27–2.61)	0.001

* *Adjusted for variables including age, sex, BMI, DM, HTN, myocardial infarction, and cancer*.

** *Adjusted for variables included in model 1 and laboratory variables including hemoglobin, serum albumin, C-reactive protein, uric acid, and eGFR CKD-EPI Cr-Cystatin C*.

*CCR, serum creatinine/cystatin C ratio; HR, hazard ratio; CI, confidence interval; CKD, chronic kidney disease*.

### Subgroup Analysis of Mortality by Age and Sex

Because age and sex were well known factors influencing cystatin C levels in previous studies, we further assessed this via age and sex subgroup analyses of the association between mortality and CCR according to renal function (Table 3).

**Table 3:** Subgroup analysis for all-cause mortality according to age and sex.

**CCR**	**Age < 65**		**Age ≥65 years**		**Male**		**Female**	
	**Adjusted HR (95% CI)**	***P***	**Adjusted HR (95% CI)**	***P***	**Adjusted HR (95% CI)**	***P***	**Adjusted HR (95% CI)**	***P***
**Non-CKD**							
1^st^ quintile	12.49 (1.59–98.24)	0.016	2.35 (0.87–6.34)	0.091	2.60 (1.15–5.87)	0.021	1.04 (0.21–5.10)	0.962
2^nd^ quintile	1 (ref)		1 (ref)		1 (ref)		1 (ref)	
3^rd^ quintile	5.99 (0.74–48.47)	0.094	1.46 (0.65–3.26)	0.358	1.43 (0.66–3.08)	0.366	0.96 (0.20–4.72)	0.961
4^th^ quintile	14.51 (1.89–111.29)	0.010	2.00 (0.97–4.14)	0.061	2.46 (1.22–4.98)	0.012	1.21 (0.27–5.34)	0.803
5^th^ quintile	21.00 (2.79–158.35)	0.003	2.29 (1.12–4.68)	0.023	3.34 (1.67–6.68)	0.001	1.41 (0.32–6.15)	0.645
**CKD**							
1^st^ quintile	1.16 (0.60–2.21)	0.660	1.05 (0.65–1.71)	0.841	1.02 (0.64–1.63)	0.943	1.46 (0.76–2.80)	0.259
2^nd^ quintile	1 (ref)		1 (ref)		1 (ref)		1 (ref)	
3^rd^ quintile	0.98 (0.43–2.22)	0.955	1.54 (0.98–2.41)	0.060	1.12 (0.71–1.78)	0.619	2.13 (1.08–4.19)	0.028
4^th^ quintile	1.57 (0.81–3.04)	0.183	1.47 (0.94–2.28)	0.089	1.76 (1.15–2.70)	0.010	1.02 (0.52–2.00)	0.959
5^th^ quintile	1.95 (1.01–3.78)	0.047	1.85 (1.18–2.89)	0.007	1.91 (1.22–3.02)	0.005	1.74 (0.93–3.26)	0.086

*All hazard ratio in this table adjusted for age, sex, BMI, DM, HTN, myocardial infarction, cancer, and laboratory variables including hemoglobin, serum albumin, C-reactive protein, uric acid, and eGFR CKD-EPI Cr-Cystatin C*.

*CCR, serum creatinine/cystatin C ratio; HR, hazard ratio; CI, confidence interval; CKD, chronic kidney disease*.

In patients younger than 65 years, the mortality risk according to CCR showed a similar pattern to that noted in all participants, which showed U-shaped risk in the non-CKD group, and elevated risk with increased CCR in the CKD group. The mortality risk in patients aged 65 years or older also showed a similar pattern to younger patients, but showed no increase in mortality risk with low CCR in the non-CKD group.

In male patients, the mortality risk according to CCR showed a similar pattern to that of all participants. However, in female patients, the mortality risk difference according to CCR was unaffected in both the CKD and non-CKD groups.

## Discussion

We discovered a U-shaped relationship between mortality and CCR in participants with normal renal function and increased mortality only in high CCR in participants with renal impairment. We found that the relationship between mortality and CCR was diminished in old age, as well as in female participants.

Two biomarkers commonly used to measure renal function, namely creatinine and cystatin C, are molecules with different characteristics. Creatinine is produced at a constant rate in muscle, whereas cystatin C is produced in all nucleated cells, and the sizes of these two markers are also different (2, 15). Cystatin C is considered a more sensitive biomarker due to the change in its concentration from the early stages of renal impairment, whereas the change in creatinine concentration is not significant in the early stages of renal impairment due to increased tubular secretion of creatinine (16, 17). Therefore, CCR is affected by the degree of renal impairment as well as several non-renal factors that affect creatinine and cystatin C levels.

In this study, the relationship between CCR and mortality according to renal function was analyzed. Some results demonstrated differences, while other results showed similar patterns between patients with normal and impaired renal function. First, a low CCR showed increased risk of mortality in the population with normal renal function, but the association with mortality decreased in the case of renal impairment. Previous studies of low CCR have suggested an association with sarcopenia, and a low CCR has been considered to be an indicator of increased risk of mortality in various diseases (9, 11, 18). A study of DM patients suggested a low CCR as a marker of sarcopenia, and reported that a low CCR lowered quality of life and increased mortality (9). Another study on patients with obstructive coronary artery disease also suggested that CCR is a surrogate marker for sarcopenia, and a lower CCR was related to higher prevalence of major adverse cardiovascular events which including all-cause mortality (11). In addition to the association with sarcopenia, the association with mortality is also considered in terms of the clinical entity named "shrunken pore syndrome”, which was reported as a consequence of the decrease in the pore size of the glomerular filtration barrier (15, 19). However, in our study, in patients with renal impairment these mortality risk changes disappeared after adjusting for nutritional and inflammatory factors and eGFR in the participants, suggesting that chronic inflammation and malnutrition accompanying renal impairment have a greater effect on mortality than CCR. Therefore, the effect of a low CCR as an indicator of sarcopenia or mortality in patients with renal impairment may not be conclusive. Other effective sarcopenia markers should be identified in these patients.

In contrast, participants with a high CCR showed consistently high mortality in both the CKD and non-CKD groups. In previous studies, low cystatin C has been shown to decrease infection resistance and exacerbate plaque growth by enhancing chronic low-grade inflammatory stimuli (20). Another study showed an association between a genetically determined decrease in cystatin C expression and increased severity of coronary artery disease (21). In regard to these findings, the increased atherosclerotic and cardiovascular risk associated with high CCR is thought to influence the mortality risk. However, there is still a lot of controversy about cystatin C and its clinical consequences, and a recent study on patients with acute illness in intensive care units showed results opposite to those found in this study (22), suggesting that cystatin C may exhibit different patterns in chronic illness compared to acute illness.

In addition, the results of our study showed that the association between CCR and mortality risk decreased in the old age and female population in the subgroup analysis, which may be due to differences in muscle mass, or hormonal effects. The differences relating to these demographic factors and renal function suggests that CCR cannot be a universal predictor of mortality in all populations.

This study has the advantage of intensively analyzing the effects of renal function on CCR, which has been neglected in many previous studies, but our study does have some limitations. This is a retrospective study and cannot reveal a causal relationship, therefore it cannot confirm whether a low or high CCR is the cause of inflammatory or sarcopenic conditions, or simply a result of such conditions. The cause of mortality could not be analyzed due to a lack of data. Additionally, our study is a single center analysis, requiring additional validation analyzes to confirm the consistency and reliability of our findings.

Along with the widespread use of assessment of renal function using cystatin C in nephrology, interest in the difference between creatinine and this new marker, and the cause of the difference, is increasing. In this study, we reported different clinical implications of discrepancies between creatinine and cystatin C levels in normal and impaired renal function. Additional research to reveal the etiology of the discrepancy between these groups is needed in the future.

## Data Availability Statement

The raw data supporting the conclusions of this article will be made available by the authors, without undue reservation.

## Ethics Statement

The studies involving human participants were reviewed and approved by the Institutional Review Board of Korea University Guro Hospital (Approval No. 2021GR0523). Written informed consent for participation was not required for this study in accordance with the national legislation and the institutional requirements.

## Author Contributions

JH: data analysis and interpretation and manuscript drafting. YS, JS, and EC: data collection. SA, GK, and YK: technical support and supervision. JK: project development, data interpretation, supervision, and manuscript editing. All authors contributed to the article and approved the submitted version.

## Funding

This work was supported and funded by Korea University Guro Hospital (KOREA RESEARCH-DRIVEN HOSPITAL) grant (No. O2000991).

## Conflict of Interest

The authors declare that the research was conducted in the absence of any commercial or financial relationships that could be construed as a potential conflict of interest.

## Publisher's Note

All claims expressed in this article are solely those of the authors and do not necessarily represent those of their affiliated organizations, or those of the publisher, the editors and the reviewers. Any product that may be evaluated in this article, or claim that may be made by its manufacturer, is not guaranteed or endorsed by the publisher.
